# Supplementary figures and images for: Role of the endothelial cell apolipoprotein E receptor 2 in modulating the effects of apoE3 and apoE4 on insulin blood-brain barrier transport
Source: PLoS One. 2026 Feb 18;21(2):e0343155. doi: 10.1371/journal.pone.0343155 (PMC12915945; doi:10.1371/journal.pone.0343155)

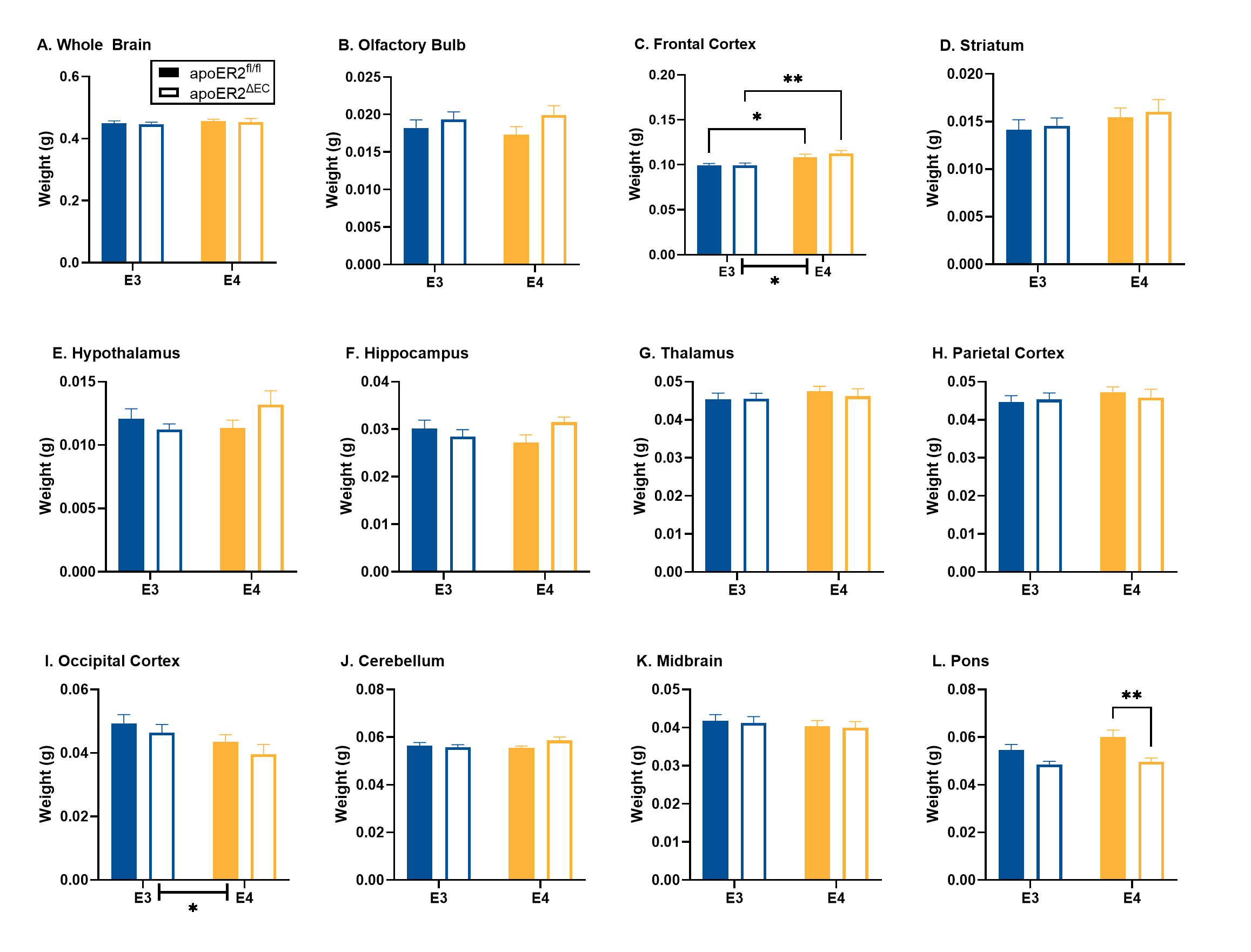

Supplement: S1 Fig — The effects of apoE isoform and endothelial apoER2 on brain region weight are graphed for A) whole brain, B) olfactory bulb, C) frontal cortex, D) striatum, E) hypothalamus, F) hippocampus, G) thalamus, H) parietal cortex, I) occipital cortex, J) cerebellum, K) midbrain, and L) pons/medulla. Means reported ± SEM. ANOVA differences are indicated by brackets between E3 vs E4. Fisher’s LSD post hoc differences are marked, *p < 0.05, **p < 0.01. Final sample sizes reflect apoE3;apoER2fl/fl n = 18, apoE3;apoER2ΔEC n = 27, apoE4;apoER2fl/fl n = 23, apoE4;apoER2ΔEC n = 12 for all regions except when outliers were removed by the ROUT method (Q = 1%) and included OB: n = 1 apoE4;apoER2fl/fl; Pons: n = 1 apoE3;apoER2fl/fl, n = 5 apoE3;apoER2ΔEC. WB: whole brain, OB: olfactory bulb, FCtx: frontal cortex, Str: striatum, Thal: thalamus, Hippo: hippocampus, Hypo: hypothalamus, PCtx: parietal cortex, OCtx: occipital cortex, CB: cerebellum. (TIF) [file pone.0343155.s001.tif]

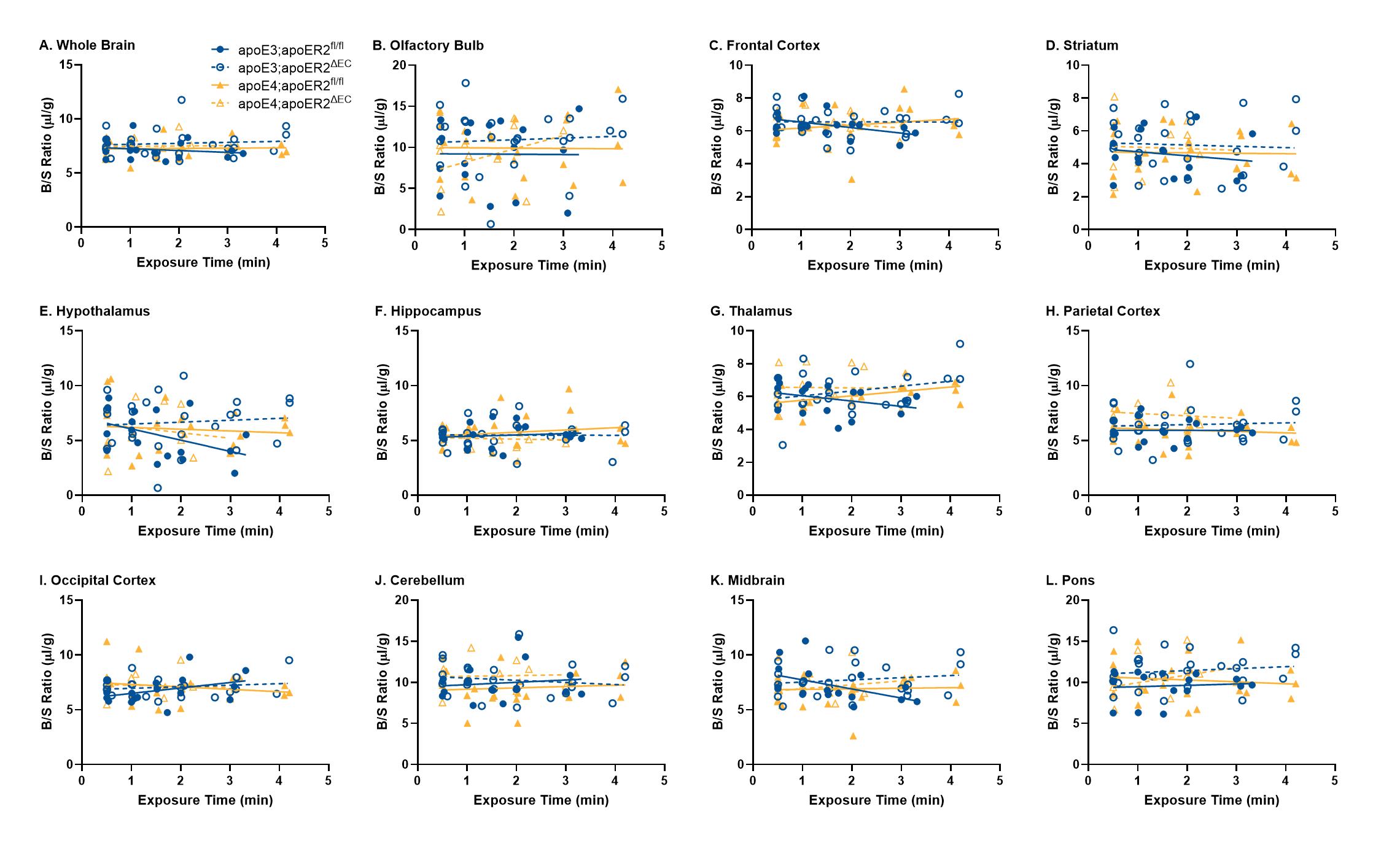

Supplement: S2 Fig — Multiple-time linear regressions for 99mTc-albumin brain/serum (B/S) ratios were plotted against exposure time for each brain region (A-L). There was no significant linear regression within each region for any group, except in the G) thalamus in which the apoE4;apoER2fl/fl group had a positive linear regression (r = 0.47, p = 0.026). Final sample sizes reflect apoE3;apoER2fl/fl n = 17, apoE3;apoER2ΔEC n = 25, apoE4;apoER2fl/fl n = 24, apoE4;apoER2ΔEC n = 11 for most regions with additional outliers removed by the ROUT method (Q = 1%) including, Frontal Cortex: n = 1 apoE3;apoER2ΔEC (n = 24 total), Thalamus: n = 1 apoE3;apoER2ΔEC n = 24 total), and n = 2 apoE4;apoER2fl/fl (n = 22 total), Occipital Cortex: n = 2 apoE3;apoER2ΔEC (n = 23 total) and n = 1 apoE4;apoER2fl/fl (n = 10 total), Pons: n = 1 apoE3;apoER2fl/fl (n = 16 total). (TIF) [file pone.0343155.s002.tif]

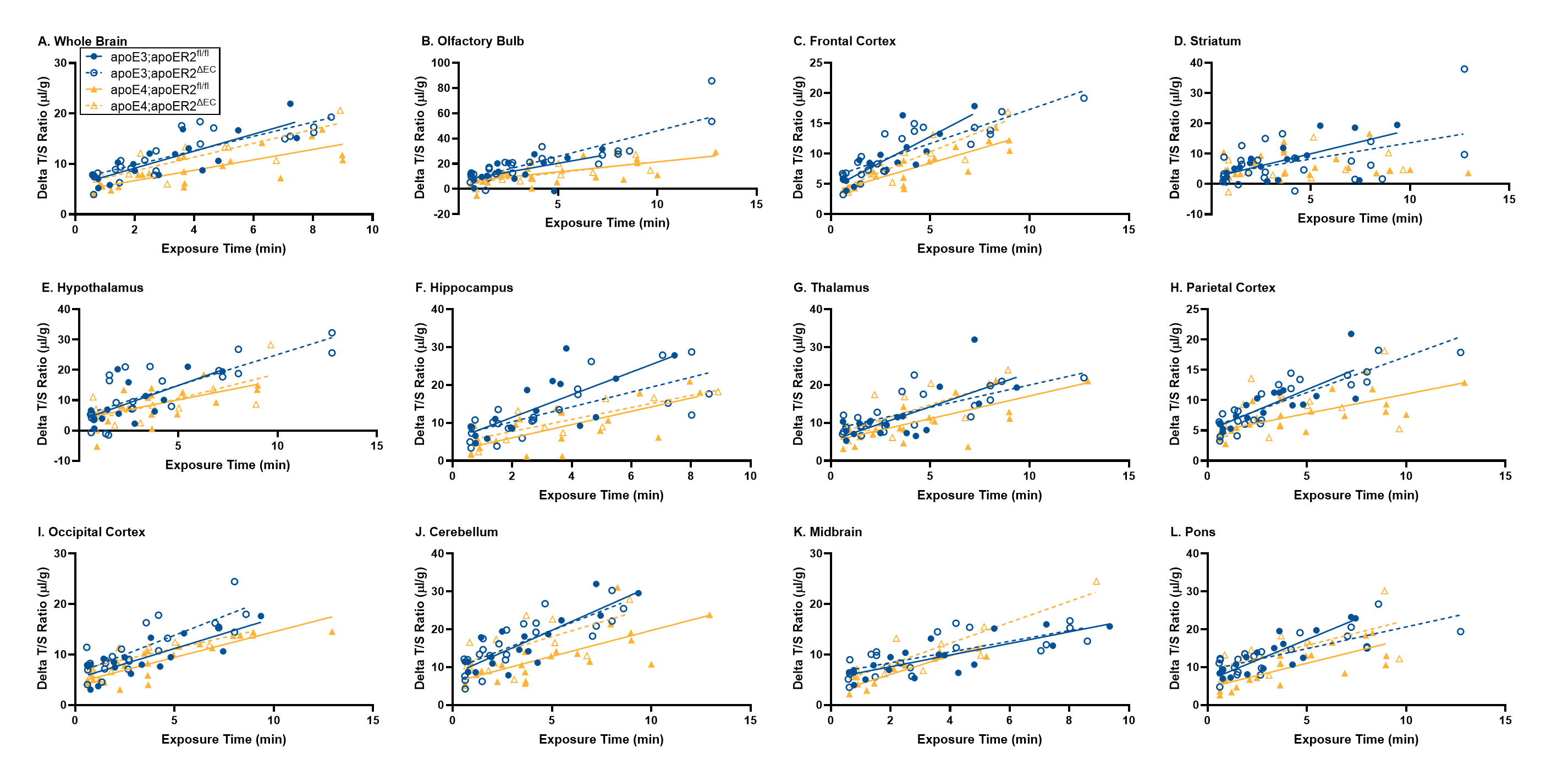

Supplement: S3 Fig — Multiple-time linear regressions for delta brain/serum (B/S) ratios were plotted against exposure time for each brain region (A-L). There was significant linear regression within each region for most groups (solid or dotted lines), except in the D) striatum in which the apoE4;apoER2fl/fl group (r = 0, p = 0.99) and apoE4;apoER2ΔEC group (r = 0.32, p = 0.31) did not have a significant linear regression (r = 0.39, p = 0.21), nor the H) parietal cortex in the apoE4;apoER2ΔEC group (r = 0.39, p = 0.21). (TIF) [file pone.0343155.s003.tif]
